# Preoperative and postoperative ultrasound guided intercostal nerve blockade on postoperative analgesia in patients undergoing video assisted thoracic surgery (VATS): a prospective randomized comparative study

**DOI:** 10.3389/fmed.2026.1825123

**Published:** 2026-09-04

**Authors:** Nan Xie, Lu Luo, Yun Zhu, Mengqi Zhu, Xin Wang

**Affiliations:** 1 Fudan University Shanghai Cancer Center, Shanghai, China; 2 Fudan University Eye Ear Nose and Throat Hospital, Shanghai, China

**Keywords:** intercostal nerve block (ICNB), lung cancer, postoperative pain, postoperative sleep quality, video-assisted thoracic surgery (VATS)

## Abstract

**Objectives:**

Ultrasound-guided intercostal nerve block (ICNB) is an effective modality for alleviating pain after video-assisted thoracic surgery (VATS). However, the optimal timing for its administration remains controversial. This study aimed to compare the analgesic efficacy of preoperative versus postoperative ICNB in patients undergoing VATS.

**Methods:**

A total of 172 adult patients undergoing VATS were randomized into two parallel groups: the pre-ICNB group (preoperative ultrasound-guided ICNB, *n* = 88) and the post-ICNB group (postoperative ultrasound-guided ICNB, *n* = 84). The primary outcome was the incidence of at least one episode of moderate-to-severe pain at rest (Numeric Rating Scale [NRS] score > 4) during the first 24 h postoperatively. Secondary outcomes included NRS scores during coughing, perioperative opioid consumption, sleep quality, and the incidence of postoperative complications.

**Results:**

The incidence of moderate-to-severe resting pain during the first 24 h postoperatively was significantly lower in the post-ICNB group than in the pre-ICNB group (11.9% vs. 28.4%, *P* < 0.05). Both static (at rest) and dynamic (during coughing) NRS scores were significantly lower in the post-ICNB group at 4, 6 and 8 h postoperatively (*P* < 0.05); however, comparable pain scores were observed upon admission to the post-anesthesia care unit (PACU) and at 2, 24, and 48 h postoperatively (*P* > 0.05). Cumulative postoperative opioid consumption at 24 and 48 h was significantly lower in the post-ICNB group (*P* < 0.01). Furthermore, patients in the post-ICNB group exhibited superior sleep quality during the first two postoperative nights (*P* < 0.01). No significant differences were observed in total (intraoperative + postoperative) opioid consumption, overall recovery quality, or surgical/anesthetic complications.

**Conclusion:**

Postoperative ICNB provides superior early postoperative analgesia, reduces postoperative opioid requirements, and improves sleep quality compared with preoperative ICNB. These findings suggest that postoperative administration is a more effective strategy for acute pain management following VATS.

**Clinical trial registration:**

https://www.chictr.org.cn/showproj.aspx?proj=183278, identifier ChiCTR2300079183.

## Introduction

Video-assisted thoracic surgery (VATS) has emerged as the standard minimally invasive approach for lung resections. Compared with traditional open thoracotomy, VATS minimizes surgical trauma, accelerates functional recovery, and shortens hospital stays (1). Nevertheless, a significant proportion of patients undergoing VATS still experience moderate-to-severe acute postoperative pain. Inadequate pain relief can impair early diaphragmatic movement, restrict coughing, and impede expectoration, thereby increasing the risk of postoperative pulmonary complications (2). Furthermore, poorly controlled acute pain is a well-established risk factor for the development of chronic post-thoracotomy pain syndrome (3, 4).

Regional nerve blocks are strongly recommended as core components of multimodal analgesia within Enhanced Recovery After Surgery (ERAS) pathways for thoracic surgery (5–7). Given that acute post-thoracic surgery pain stems primarily from mechanical stimulation and injury to the intercostal nerves, intercostal nerve blockade (ICNB) has gained widespread adoption (8). Accumulating evidence supports the efficacy and safety of ICNB in enhancing postoperative analgesia and patient satisfaction (9, 10). However, the duration of a single-injection preoperative ICNB is often limited, typically lasting no more than 4 h during dynamic activity (such as coughing) and 6 h at rest (6, 11). Consequently, maximizing the clinical efficacy of ICNB remains a key objective. Our previous work demonstrated that rescue ICNB administered in the post-anesthesia care unit (PACU) rapidly and effectively alleviated acute pain in patients undergoing esophagectomy (12). However, the optimal procedural timing–preoperative versus postoperative administration–has not been fully elucidated or standardized for routine thoracoscopic surgery.

Therefore, this prospective, randomized, single-blind study was designed to compare the effects of preoperative and postoperative ultrasound-guided ICNB on pain scores, analgesic duration, perioperative opioid consumption, sleep quality, and complication rates in patients undergoing VATS, aiming to identify the optimal timing to refine acute pain management.

## Methods

### Study design and ethics

This randomized, evaluator-blinded, parallel-group trial was conducted at Fudan University Shanghai Cancer Center to compare the clinical efficacy of preoperative versus postoperative ultrasound-guided ICNB. The study protocol received formal approval from the Institutional Ethics Committee (Approval No.: 2105236-13). Written informed consent was obtained from all participants prior to enrollment. The trial was prospectively registered in the Chinese Clinical Trial Registry (Registration No.: ChiCTR2300079183; Date of registration: December 27, 2023).

### Patient recruitment and randomization

**FIGURE 1:**
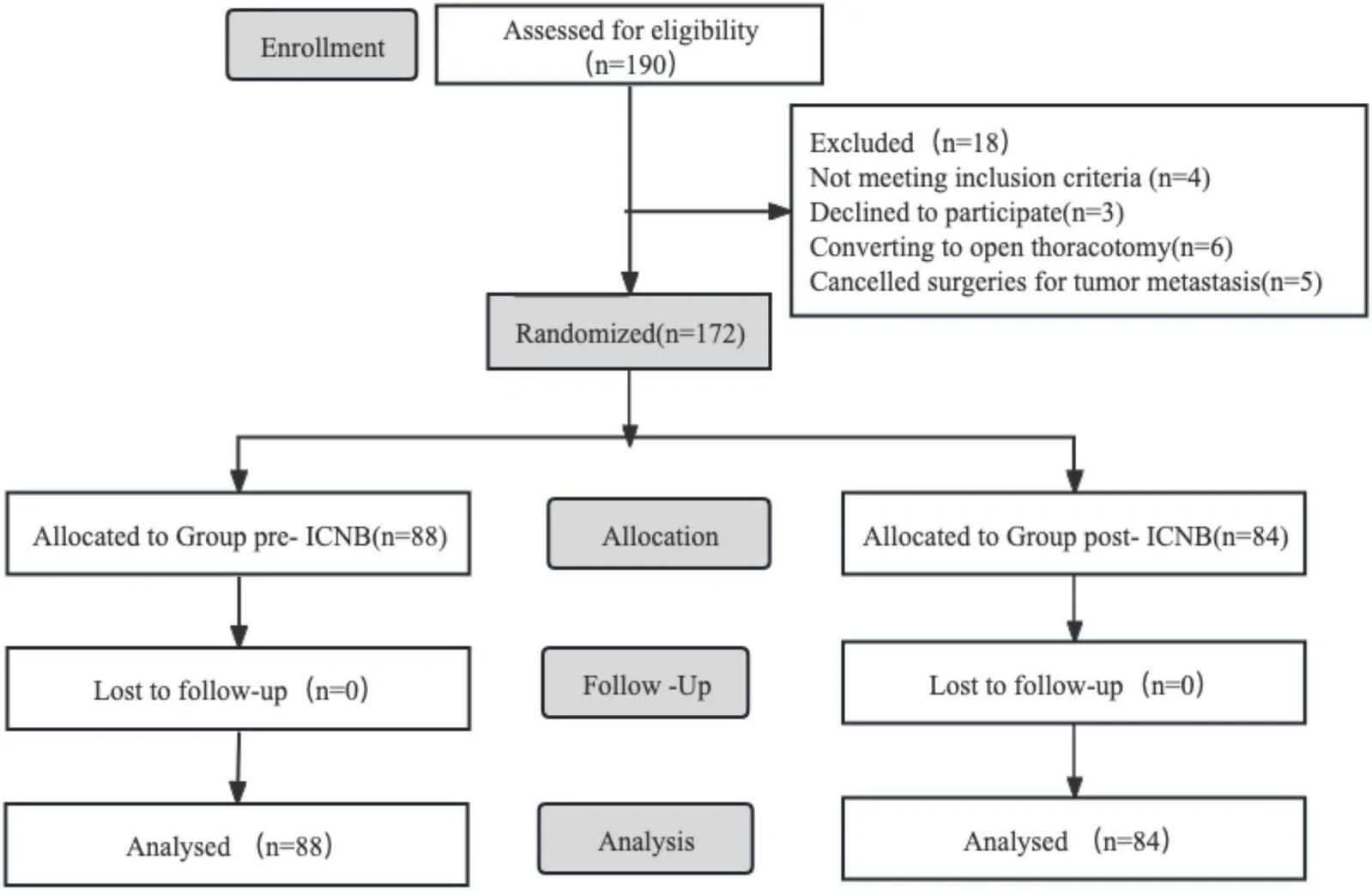
CONSORT flow diagram. CONSORT indicates Consolidated Standards of Reporting Trials.

From December 2023 to June 2024, patients aged 18–70 years with an American Society of Anesthesiologists (ASA) physical status of I or II who were scheduled for VATS lobectomy or sleeve lobectomy were screened for eligibility. The exclusion criteria included: a known allergy to local anesthetics; chronic use of analgesics or sedatives; severe psychiatric or psychological disorders; coagulopathy; anatomical abnormalities of the spine or thoracic wall; and pre-existing sleep disorders requiring pharmacological intervention within the past month (e.g., severe insomnia or sleep apnea). The CONSORT flow diagram is shown in Figure 1.

Eligible participants were randomized into the pre-ICNB or post-ICNB group at a 1:1 ratio via a computer-generated randomization sequence. Group assignments were concealed in sequentially numbered, opaque, sealed envelopes, which were opened 1 h before anesthesia induction by an independent researcher. Except for the attending anesthesiologists performing the nerve blocks, all patients, ward nurses, postoperative follow-up assessors, and statisticians were strictly blinded to group allocation. All surgical procedures and nerve blocks were executed by the same dedicated team of thoracic surgeons and anesthesiologists to ensure technical consistency.

### Anesthetic managements

No preoperative sedative medications were administered. Upon arrival in the operating room, standard monitoring was established, including electrocardiography, pulse oximetry, bispectral index (BIS), and invasive arterial blood pressure.

After 5 min of preoxygenation with 100% oxygen, anesthesia induction was performed using target-controlled infusion (TCI) of propofol (target plasma concentration: 3–5 μg/mL), sufentanil (0.03 μg/kg), and rocuronium (0.6 mg/kg). Dexamethasone (4 mg) was administered intravenously at induction, and granisetron (3 mg) was given near the end of surgery to prevent postoperative nausea and vomiting (PONV).

Following endotracheal intubation with a double-lumen tube, lung-protective ventilation was initiated: tidal volume of 4–6 mL/kg, positive end-expiratory pressure (PEEP) of 5 cmH_2_O, and intermittent alveolar recruitment maneuvers during one-lung ventilation. The fraction of inspired oxygen was titrated to maintain peripheral oxygen saturation (SpO2) ≥ 95%. General anesthesia was maintained with infusions of propofol and remifentanil via TCI, titrated to maintain a BIS value between 40 and 60. Additional boluses of sufentanil (0.1–0.2 μg/kg) and rocuronium (0.15–0.3 mg/kg every 30 min) were administered based on hemodynamic parameters and surgical duration. Intraoperative hypotension (mean arterial pressure < 20% of baseline) was treated with norepinephrine or ephedrine, and bradycardia (heart rate < 45 beats/min) was managed with 0.5 mg of atropine.

### Intercostal nerve block technique

**FIGURE 2:**
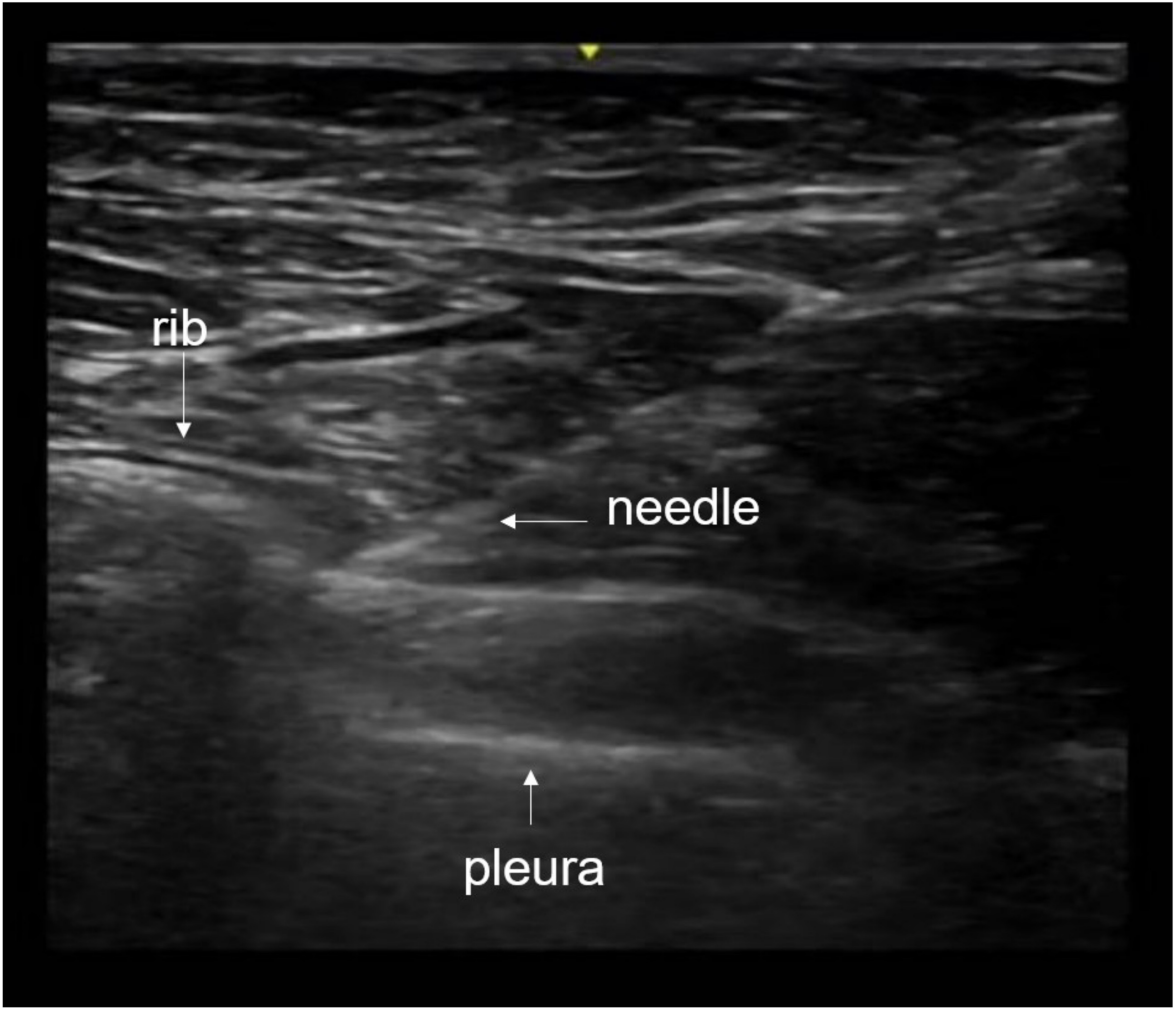
Ultrasound-guided intercostal nerve block.

After endotracheal intubation, patients in the pre-ICNB group were placed in the lateral decubitus position. Before skin disinfection, a linear array transducer (Venue40, GE Healthcare, WI, USA) with a frequency range of 6–13 MHz was placed in a longitudinal parasagittal orientation to identify the target ribs, internal intercostal muscles, external intercostal muscles and pleura (Figure 2). After sterilization of the injection site, an 18-gauge, 10-cm needle (Stimuplex A, Braun, Melsungen, Germany) connected to a 20 mL syringe was advanced toward the inferior margin of the target ribs using the in-plane technique under real-time ultrasound guidance. Needle tip position was verified by injection of 0.2–0.5 mL normal saline. Following negative aspiration for blood and air, 3 mL of 0.25% ropivacaine (Naropin^®^, AstraZeneca, Switzerland) was injected into each of the upper and lower two target intercostal spaces adjacent to the parietal pleura. Visualized downward displacement of the parietal pleura confirmed a successful block. In the post-ICNB group, the ICNB was performed at the end of the surgical procedure. In this study, anesthesia management follows standardized protocols to maintain procedural consistency and ICNB procedures were performed by the same two experienced anesthesiologists.

Upon completion of surgery, propofol and remifentanil infusions were discontinued. Patients were transferred to the PACU, where neuromuscular blockade was reversed with sugammadex (2–4 mg/kg) prior to tracheal extubation. Patients were discharged to the ward when their modified Aldrete score was 9 (13).

### Postoperative analgesia and outcome assessments

All patients received standardized patient-controlled intravenous analgesia (PCIA) formulated with sufentanil (0.5 mcg/ml) and flurbiprofen axetil (0.75 mg/mL). The PCIA pump settings were programmed to a background infusion rate of 3.5 mL/h, a demand bolus of 4 mL, and a lockout interval of 15 min. Postoperative pain at rest and during coughing was evaluated by a blinded outcome assessor using an 11-point Numeric Rating Scale (NRS; 0 = no pain, 10 = worst imaginable pain) at predetermined intervals: upon PACU admission, and at 2, 4, 6, 8, 12, 24, and 48 h postoperatively. If the resting NRS score was ≥4, intravenous morphine (50 μg/kg) was administered as a rescue analgesic.

#### Outcome measures

The primary outcome was the cumulative incidence of at least one recorded episode of moderate-to-severe resting pain (NRS score > 4) during the first 24 h postoperatively (14).

Secondary outcomes included time-course NRS pain scores, total and interval opioid consumption (expressed as morphine milligram equivalents, MME), rescue analgesic frequency, sleep quality, and perioperative complications. The Pittsburgh Sleep Quality Index (PSQI) was used to assessed preoperative sleep quality, and the Chinese version of the Richards Campbell Sleep Questionnaire (RCSQ) was used to assess postoperative nighttime sleep quality. The PSQI consists of 19 items divided into 7 components, and higher scores indicate poorer sleep quality (15). The RCSQ is used to examine rapid changes in sleep quality and is divided into five dimensions: sleep depth, sleep latency, awakening, return to sleep, and sleep quality (16).

### Statistical analysis

#### Calculation of sample size

Sample size calculation was based on pilot trial data (10 patients per group), where the incidence of moderate-to-severe pain at rest during the first 24 h postoperatively was 30% in the pre-ICNB group and 10% in the post-ICNB group. To achieve a statistical power of 80% with a two-sided significance level of 5% (α = 0.05), a minimum of 59 patients per group was required (PASS software, version 15.0). Accounting for a predicted 10% dropout rate, the minimum enrollment was set at 132 patients. To further improve the statistical power of the study and avoid potential biases caused by insufficient sample size and accidental data loss, we appropriately expanded the enrollment scale on the basis of the calculated minimum sample size. Finally, a total of 172 eligible patients were enrolled and included in the final statistical analysis.

SPSS 25.0 for Windows (SPSS Inc., Chicago, IL, USA) software was used for all the statistical analyses. The Kolmogorov–Smirnov test was used to test the normality of the data. Normally distributed data are presented as means (standard deviations) and were compared using the independent samples *t*-test. Non-normally distributed data are presented as rates (percentages) or medians (interquartile ranges) and were compared using the Mann–Whitney U test. Categorical variables were analyzed via the chi-square test or Fisher’s exact test. A *P*-value < 0.05 indicated statistical significance.

## Results

A total of 190 patients were assessed for eligibility. Eighteen patients were excluded: 4 failed to meet inclusion criteria, 3 declined participation, 6 required conversion to open thoracotomy, and 5 had surgeries canceled due to intraoperative detection of distant tumor metastasis. Ultimately, 172 patients completed the study, with 88 patients in the pre-ICNB group and 84 patients in the post-ICNB group (Figure 1). Baseline demographic variables, clinical characteristics, American Society of Anesthesiologists (ASA) status, comorbidities, and surgical parameters (including duration and resection type) were comparable between the two groups (Table 1; *P* > 0.05).

**TABLE 1:** Patient demographics and clinical characteristics.

Characteristic	Group pre- ICNB (*n* = 88)	Group post- ICNB (*n* = 84)	*P*-value
Age	55.57 ± 9.28	56.24 ± 9.04	0.54
Gender (male/female)	32/56	33/51	0.69
Body mass index.	24.96 ± 7.14	23.87 ± 3.12	0.20
Marital status (married /single)	69/19	63/21	0.60
ASA status (I/II)	30/58	32/52	0.59
Left/right	37/51	33/51	0.71
Diabetes	11	11	1.00
Hypertension	30	19	0.10
Pulmonary disease	7	6	1.00
Non-smoker/ smoker	18/70	19/65	0.73
Surgery duration (min)	106.94 ± 37.31	105.27 ± 28.18	0.34
VATS type: lobectomy /segmentectomy	37/51	46/38	0.10

ASA, American society of Anesthesiologists. Values are expressed as mean ± SD or number.

The incidence of moderate-to-severe pain at rest within the first 24 h postoperatively (the primary outcome) was significantly lower in the post-ICNB group than in the pre-ICNB group (11.9% vs. 28.4%, *P* < 0.05).

For dynamic pain scores (during coughing), RM-ANOVA demonstrated significant main effects for group (F(group) = 141.746, *P* < 0.001), time (F(time) = 284.434, *P* < 0.001), and a significant group-by-time interaction (F(group × time) = 74.218, *P* < 0.001). *Post hoc* pairwise comparisons indicated that pain scores during coughing were significantly lower in the post-ICNB group than in the pre-ICNB group at 4, 6, 8, and 12 h postoperatively (Table 2, *P* < 0.001). The most pronounced difference occurred at 8 h postoperatively, where the pre-ICNB group experienced a severe pain peak compared to the post-ICNB group (5.14 ± 0.90 vs. 3.39 ± 0.58, *P* < 0.001). No significant intergroup differences were observed upon PACU admission or at 2, 24, and 48 h postoperatively (*P* > 0.05).

**TABLE 2:** Comparison of numerical rating scale mean pain score at coughing up to postoperative 48 h.

Time point	Group pre- ICNB (*n* = 88)	Group post- ICNB (*n* = 84)	*P*-value
PACU	2.15 ± 0.36	2.13 ± 0.34	0.753
Postoperative 2 h	2.43 ± 0.52	2.38 ± 0.60	0.553
Postoperative 4 h	3.13 ± 0.67	2.61 ± 0.60	0.000
Postoperative 6 h	4.78 ± 0.82	3.08 ± 0.59	0.000
Postoperative 8 h	5.14 ± 0.90	3.39 ± 0.58	0.000
Postoperative 12 h	3.66 ± 1.03	3.17 ± 0.60	0.000
Postoperative 24 h	2.97 ± 0.51	2.94 ± 0.39	0.716
Postoperative 48 h	2.73 ± 0.56	2.67 ± 0.55	0.474
F(group) = 141.746, *P* = 0.000			
F(time) = 284.434, *P* = 0.000			
F(group × time) = 74.218, *P* = 0.000			

Values are expressed as mean ± SD. PACU, post-anesthesia care unit.

For static pain scores (at rest), RM-ANOVA similarly revealed significant main effects for group (F(group) = 25.292, *P* < 0.001), time (F(time) = 103.407 = 103.407, *P* < 0.001), and a significant interaction (F(group × time) = 13.198, *P* < 0.001). Resting pain scores were significantly lower in the post-ICNB group than in the pre-ICNB group at 4, 6, and 8 h postoperatively (Table 3, *P* < 0.001). The peak intergroup difference was observed at 6 h postoperatively (2.64 ± 1.14 in the pre-ICNB group vs. 1.69 ± 0.71 in the post-ICNB group, *P* < 0.001). No significant differences were noted at PACU admission or at 2, 12, 24, and 48 h postoperatively (*P* > 0.05).

**TABLE 3:** Comparison of numerical rating scale mean pain score at rest up to postoperative 48 h.

Time point	Group pre- ICNB (*n* = 88)	Group post- ICNB (*n* = 84)	*P*-value
PACU	1.23 ± 0.42	1.13 ± 0.34	0.100
Postoperative 2 h	1.44 ± 0.52	1.38 ± 0.4	0.421
Postoperative 4 h	1.77 ± 0.69	1.40 ± 0.49	0.000
Postoperative 6 h	2.64 ± 1.14	1.69 ± 0.71	0.000
Postoperative 8 h	2.93 ± 1.51	2.14 ± 0.79	0.000
Postoperative 12 h	2.44 ± 1.05	2.23 ± 0.63	0.100
Postoperative 24 h	2.23 ± 0.58	2.08 ± 0.59	0.108
Postoperative 48 h	1.42 ± 0.64	1.39 ± 0.62	0.774
F(group) = 25.292, *P* = 0.000			
F(time) = 103.407, *P* = 0.000			
F(group × time) = 13.198, *P* = 0.000			

Values are expressed as mean ± SD. PACU, post-anesthesia care unit.

Perioperative opioid consumption details are summarized in Table 4. There was no statistically significant difference in total morphine consumption between the two groups, as measured by the morphine milligram equivalent (MME) (114.57 ± 11.97 mg vs. 114.14 ± 6.79 mg, *p* > 0.05). However, interval analysis revealed that intraoperative MME consumption was significantly lower in the pre-ICNB group (31.60 ± 5.48 vs. 37.01 ± 3.82 mg, *P* < 0.05). Conversely, the post-ICNB group demonstrated significantly lower cumulative opioid requirements at both 24 h postoperatively (37.74 ± 4.34 mg vs. 43.59 ± 6.62 mg, *P* < 0.01) and 48 h postoperatively (77.13 ± 6.57 mg vs. 82.97 ± 7.33 mg, *P* < 0.01). Additionally, the mean time to the first required postoperative rescue opioid dose was significantly prolonged in the post-ICNB group (151.76 ± 33.19 min vs. 134.69 ± 40.83 min, *P* < 0.01).

**TABLE 4:** Opioids consumption at different time intervals.

Outcome variable	Group pre-ICNB (*n* = 88)	Group post-ICNB (*n* = 84)	*P*-value
Total intraoperative morphine (MME)	31.60 ± 5.48	37.01 ± 3.82	<0.01
Total morphine (MME) at postoperative 24 h	43.59 ± 6.62	37.74 ± 4.34	<0.01
Total morphine (MME) at postoperative 48 h	82.97 ± 7.33	77.13 ± 6.57	<0.01
Total morphine (MME)	114.57 ± 11.97	114.14 ± 6.79	0.77
Time to first opioid^a^	134.69 ± 40.83	151.76 ± 33.19	<0.01

Values are expressed as mean ± SD. MME, morphine milligram equivalent.

^a^The time from the first intravenous injection of opioid drugs to the start of surgery (min).

Preoperative sleep quality evaluated via the PSQI was comparable between groups (*P* = 0.19). However, total RCSQ scores were significantly higher (indicating better sleep quality) in the post-ICNB group during the first 24 h (63.18 ± 5.61 vs. 58.87 ± 3.27, *P* < 0.01) and from 24 to 48 h postoperatively (70.88 ± 4.82 vs. 67.95 ± 3.17, *P* < 0.01; Table 5). Domain-specific analysis showed that within the first 24 h, patients in the post-ICNB group scored significantly higher in falling asleep, frequency of awakening, return to sleep, and overall sleep quality (*P* < 0.05). During the 24–48 h interval, the post-ICNB group maintained significantly superior scores in the falling asleep and sleep quality domains (Supplementary Table 1).

**TABLE 5:** Sleep quality compared at multiple points.

Outcome variable	Pre- ICNB group (*n* = 88)	Post- ICNB group (*n* = 84)	*P*-value
PSQI before surgery	6. 48 ± 0.84	6.3 0 ± 0.94	0.19
RCSQ from 0 to 24 h post-operation	58.87 ± 3.27	63.18 ± 5.61	<0.01
RCSQ from 24 to 48 h post-operation	67.95 ± 3.17	70.88 ± 4.82	<0.01

Values are expressed as mean ± SD.

### Recovery profiles and adverse events

No significant differences were observed between the pre-ICNB and post-ICNB groups regarding the incidences of postoperative hypoxemia, dizziness, PONV, shivering, or hypotension (Table 6; *P* > 0.05). Furthermore, recovery time in the PACU, total length of PACU stay, and duration of chest tube drainage were statistically similar between the two cohorts (*P* > 0.05). No block-related complications, such as pneumothorax, the local anesthetic systemic toxicity (LAST), or hematoma, occurred in either group.

**TABLE 6:** Postoperative profiles and adverse events.

Outcome variable	Pre- ICNB group (*n* = 88)	Post- ICNB group (*n* = 84)	*P*-value
Hypoxemia (SpO_2_ < 90%)	3 (4.5)	3 (4.5)	1.00
Dizzy	11 (12.5)	9 (10.7)	0.72
PONV	12 (14.3)	10 (11.9)	0.73
Shivering	5 (7.6)	4 (6.1)	1.00
Hypotension	0	0	1.00
Recovery time in PACU (min)	22.74 ± 1.30	23.05 ± 2.16	0.33
Length of stay in PACU (min)	48.31 ± 5.59	49.77 ± 4.06	0.09
Chest tube duration (d)	2.0 (1.0–5.0)	2.0 (1.0–5.0)	0.77

PONV, postoperative nausea and vomiting; PACU, post-anesthesia care unit. Values are expressed as mean ± SD or *N* (*n* %) or Median (interquartile range).

## Discussion

This prospective, randomized, single-blind comparative trial demonstrated that compared with a preoperative block, an ultrasound-guided ICNB performed postoperatively for VATS provides significantly better early postoperative analgesia, reduces early postoperative opioid reliance, delays the need for first rescue analgesics, and improves sleep quality during the initial 48 h.

Although VATS might cause minimal operative trauma, a substantial proportion of patients, up to 38%, experience significant postoperative acute pain, with 25% of patients continuing to experience chronic pain 6 months postoperatively (11, 17). Postoperative pain may lead to a reduction in tidal volume and an increase in the respiratory rate, hinder spontaneous deep breathing, and worsen inadequate alveolar ventilation (18). Additionally, discomfort from the chest incision and drainage tube can decrease the patient’s ability to take deep breaths, causing them to be hesitant to cough or have a weak cough, which leads to reduced lung ventilation and failed mucus clearance. Therefore, adequate pain management to improve respiratory function and reduce the risk of atelectasis and other postoperative pulmonary complications is imperative.

Thoracic epidural analgesia (TEA) is the reference standard for postoperative pain management following thoracoscopic surgery (5). However, TEA may lead to hemodynamic instability and urinary retention, as well as several procedure-related complications, such as epidural hematoma and infection. Moreover, it has a substantial failure rate of 30% and necessitates patient sobriety and cooperation, thereby imposing psychological stress on patients (19, 20). Thus, the use of TEA for postoperative pain management in VATS is controversial.

Following thoracic surgery, ICNB has a lower failure rate than TEA has and has minimal influence on respiratory function. As pain after VATS is caused mainly by intercostal nerve injury and an indwelling thoracic drainage tube, ICNB can effectively interrupt the transmission of noxious stimuli, thereby assisting in the prevention of nerve ending activation and central sensitization, consequently leading to a notable reduction in postoperative pain (21). Compared with no nerve block, a single ICNB has been shown to greatly decrease pain and the need for rescue analgesics (22). Moreover, patients who underwent intraoperative ICNB had lower postoperative opioid consumption and exhibited fewer postoperative opioid-related side effects than patients who received intravenous opioids (12).

Considering that pain is relatively intense within the first 24 h after VATS, determining whether the optimal time for an ICNB is before or after surgery to improve analgesic efficiency is clinically important (22). However, this issue has been addressed in only a few studies. Lan et al compared the effects of preoperative and postoperative ICNB, and the results revealed no significant difference between the two groups in terms of perioperative pain management, which was not consistent with our results (23). Our study revealed that both resting and coughing NRS pain scores were significantly lower in the post-ICNB group compared with the pre-ICNB group at 4 h, 6 h, 8 h, and 12 h within the first 12 postoperative hours. For resting pain, the maximal intergroup difference appeared at postoperative 6 h, while the largest disparity in coughing dynamic pain scores was observed at postoperative 8 h. The differences in the results between the Lan et al study and our study may be related to the use of local anesthetics, ICNB operators and blocking methods; for example, the ICNB was performed by an anesthesiologist under the guidance of ultrasonography in our study, whereas the ICNB was performed by a surgeon during VATS in their study (23).

In this study, the pain scores at rest and during coughing were low during the first 4 h postoperatively, peaked between 6 and 24 h postoperatively, and gradually decreasing between 24 and 48 h postoperatively. In the early postoperative period (0–12 h), the pain scores in the post-ICNB group were lower than those in the pre-ICNB group. The NRS scores in the post-ICNB group were numerically lower than those in the pre-ICNB group 24–48 h after surgery; however, the differences were not statistically significant. In addition, total analgesic consumption within 48 h following surgery was notably lower in the post-ICNB group than in the pre-ICNB group. The higher NRS score and greater analgesic use observed in the pre-ICNB group may be attributed to the diminishing effects of ICNB performed before surgery. In addition, the ropivacaine used in this study is a long-acting local anesthetic, with an analgesic effect lasting for 6 h (22). The average duration of VATS was approximately 100 min, which elucidates the pain relief and low NRS scores observed from the end of surgery up to 4 h postoperatively in both groups, which is consistent with the findings of a study by Sanjay et al.(24). They reported that the effect of ICNB with 0.25% bupivacaine could last for 4 h after thoracotomy.

The optimal timing for performing a nerve block is debated in the literature. A randomized controlled study comparing the effects of the timing of the block on postoperative outcomes revealed that a transversus abdominis plane blockade after surgery significantly decreased postoperative opioid consumption, pain scores at rest and during coughing, and PONV incidence (25). However, Byrne et al. reported that, compared with postoperative perineural stump catheter placement for sciatic nerve block, preoperative sciatic nerve block did not reduce the incidence of phantom limb pain at 5 days, 6 months, or 12 months after knee amputation; moreover, it has analgesic advantages only in the early period after surgery (26).

Patients experience the intense pain caused by VATS for the entire 24 h after the procedure, especially during the first night after surgery (27). As a result, patients experience difficulty falling and staying asleep (28). In this study, the sleep quality in both groups decreased during the first 48 h postoperatively, indicating a significant correlation between sleep and pain scores. Poor sleep quality may increase sensitivity to pain on subsequent days, leading to stronger pain sensations and greater analgesic use. Nevertheless, daytime pain intensity and opioid usage can also affect nighttime sleep quality (27, 29). Compared with patients in the pre-ICNB group, patients in the post-ICNB group had better sleep quality during the first 24 h postoperatively, which may be related to their lower pain scores and little to no need for rescue analgesics. Although the patients in the post-ICNB group continued to have better sleep quality than those in the pre-ICNB group had during the first 24–48 h after surgery, the differences between the two groups were smaller. This is potentially attributed to the administration of opioids via a PCIA pump, which may effectively manage pain and improve sleep quality.

Consistent with previous results, our study revealed that an ICNB can reduce the incidences of postoperative hypoxemia and PONV (2–4). In addition, there were no complications attributable to ICNB, indicating that ultrasound-guided ICNB can safely reduce postoperative pain. Moreover, since low-concentration local anesthetics predominantly target the superficial layers of nerve clusters, impeding the transmission of sensory nerves while having a minor impact on motor nerves, none of the patients in our study experienced respiratory depression. Therefore, this differential sensory and motor blockade from low-concentration ropivacaine is advantageous for individuals undergoing thoracoscopy procedures.

This study has several limitations. First, we were unable to be blinded to the group assignment due to the study’s design. There was only one preoperative monitoring nighttime record, and changes in the environment may lead to sleep changes, known as the first night effect (FNE) (30). Therefore, the so-called normal observed by the PSQI before surgery may be affected. In addition, it is impossible to guarantee consistent postoperative sleep environments for all patients (such as the number of people in the ward, temperature, and absolute quietness). Moreover, the study included only a postoperative assessment at 48 h after surgery on the basis of several studies suggesting that postoperative pain peaks during this period, indicating a need for a longer follow-up period to assess the impact on long-term outcomes.

## Conclusion

Compared with preoperative ICNB, postoperative ICNB yields improved early postoperative analgesic effects, reduced postoperative opioid consumption, and better postoperative sleep quality in patients undergoing VATS. This technique can be prioritized for postoperative pain management after VATS.

## Data Availability

The original contributions presented in this study are included in this article/Supplementary material, further inquiries can be directed to the corresponding author.
