# Supplementary material for: Preoperative and postoperative ultrasound guided intercostal nerve blockade on postoperative analgesia in patients undergoing video assisted thoracic surgery (VATS): a prospective randomized comparative study
Source: Front Med (Lausanne). 2026 Sep 4;13:1825123. doi: 10.3389/fmed.2026.1825123 (PMC13585723; doi:10.3389/fmed.2026.1825123)
Supplement: Supplementary file 1 [file Table_1.docx]

Supplemental Table1 The scores in each domain of Richards Campbell Sleep Questionnaire

| Time point | pre- ICNB Group (n = 88) | post- ICNB Group (n = 84) | *P* value |
| --- | --- | --- | --- |
| RCSQ from 0-24 h post-operation |  |  |  |
| Sleep depth ^a^ | 50.13 ± 5.22 | 51.44± 5.92 | 0.12 |
| Falling asleep ^b^ | 61.97 ± 4.25 | 68.25 ± 7.61 | < 0.01 |
| Awakening ^c^ | 60.43 ± 4.28 | 62.79 ± 7.08 | 0.01 |
| Return to sleep ^d^ | 61.58 ± 4.62 | 68.87 ± 6.88 | < 0.01 |
| Sleep quality ^e^ | 60.24 ± 4.53 | 64.56 ± 7.35 | < 0.01 |
| RCSQ from 24-48 h post-operation |  |  |  |
| Sleep depth | 63.13 ± 5.22 | 62.50 ± 5.67 | 0.45 |
| Falling asleep | 62.70 ± 4.14 | 74.20 ± 6.40 | < 0.01 |
| Awakening | 70.61 ± 4.73 | 71.79 ± 7.08 | 0.21 |
| Return to sleep | 72.42 ± 4.48 | 73.01 ± 6.17 | 0.48 |
| Sleep quality | 70.88 ± 4.41 | 72.78 ± 6.77 | 0.02 |

^a^ Each of the following domain was assessed by using a 100-mm visual analog scale in which a higher score is better. Scores of a question: “My sleep last night was: Deep sleep …Light sleep”

^b^ Scores of a question: “Last night, the first time I got to sleep, I: Fell asleep almost immediately… Just never could fall asleep”.

^c^ Scores of a question: “Last night, I was: Awake very little… Awake all night”.

^d^ Scores of a question: “Last night, when I woke up or was awakened, I: Got back to sleep immediately… Couldn’t get back to sleep”.

^e^ Scores of a question: “I would describe my sleep las night as: A good night’s sleep… A bad night’s sleep”.

^f^ Scores of a question: “I would describe the noise level last night as: Very quiet… Very noisy”.
